# Sex differences in the work of breathing during exercise are independent of forced vital capacity in healthy adults

**DOI:** 10.1113/EP092586

**Published:** 2025-05-05

**Authors:** Gracie O. Grift, Jack R. Dunsford, Jasvir K. Dhaliwal, Paolo B. Dominelli, Yannick Molgat‐Seon

**Affiliations:** ^1^ Department of Kinesiology and Applied Health University of Winnipeg Winnipeg Manitoba Canada; ^2^ Centre for Heart and Lung Innovation The University of British Columbia and St. Paul's Hospital Vancouver British Columbia Canada; ^3^ Department of Kinesiology and Health Sciences University of Waterloo Waterloo Ontario Canada; ^4^ Faculty of Kinesiology University of Calgary Calgary Alberta Canada

**Keywords:** airways, incremental exercise, respiratory mechanics, respiratory muscles

## Abstract

During exercise, females have a higher work of breathing (*Ẇ*_B_) than males for a given minute ventilation (${\dot{V}}_{E}$) ≥ 50–60 L min^−1^, presumably due to sex differences in airway size. However, on average, males have greater forced vital capacity (FVC) than females, and the confounding effect of FVC on sex differences in *Ẇ*_B_ is unknown. To determine the effects of FVC and sex on *Ẇ*_B_ during exercise in healthy adults, 30 healthy adults (15 males, 15 females) completed spirometry and an incremental cycle exercise test to exhaustion. Throughout exercise, *Ẇ*_B_ was calculated based on oesophageal pressure and open‐circuit spirometry. The *Ẇ*_B_–${\dot{V}}_{E}$ relationship was compared between the sexes across all participants and in seven males and seven females matched for FVC and age. Across all participants, FVC had no effect on the *Ẇ*_B_–${\dot{V}}_{E}$ relationship (*P *= 0.323), and females had a higher *Ẇ*_B_ than males at a ${\dot{V}}_{E}$ of 50 (*P *= 0.030), 60 (*P *= 0.023), 70 (*P *= 0.021) and 80 L min^−1^ (*P *= 0.020). At a ${\dot{V}}_{E}$ of 35 ± 2 L min^−1^, FVC was not associated with *Ẇ*_B_ (*r* ^2 ^= 0.042, *P *= 0.278). Conversely, at a ${\dot{V}}_{E}$ of 70 ± 5 L min^−1^, FVC was associated with *Ẇ*_B_ across all participants (*r* ^2 ^= 0.164, *P *= 0.026), but not within each sex (males: *r* ^2 ^= 0.077, *P *= 0.317; females: *r* ^2 ^= 0.011, *P *= 0.714). In the males and females matched for FVC and age, females had a higher *Ẇ*_B_ than males at a ${\dot{V}}_{E}$ of 60 (*P *= 0.049), 70 (*P *= 0.019), 80 (*P *= 0.020) and 90 L min^−1^ (*P *= 0.014). Overall, our findings indicate that sex differences in *Ẇ*_B_ during exercise are not influenced by male–female differences in FVC.

## Abstract (highlights)

- **What is the central question of this study?**
  Do sex differences in the work of breathing (*Ẇ*_B_) during exercise persist after accounting for sex differences in forced vital capacity (FVC)?
- **What is the main finding and its importance?**
  After accounting for FVC statistically and by matching males and females for FVC and age, females had a higher *Ẇ*_B_ during exercise than their male counterparts, suggesting that sex differences in *Ẇ*_B_ during exercise are independent of FVC. Our findings provide additional support for the notion that sex differences in airway size influence *Ẇ*_B_ during exercise in healthy adults.

## 1 INTRODUCTION

The work of breathing (*Ẇ*_B_) represents the mechanical work performed by the respiratory muscles during spontaneous breathing needed to generate a given minute ventilation (${\dot{V}}_{E}$). The total *Ẇ*_B_ can be subdivided into two main components: viscoelastic work and resistive work (Dominelli & Sheel, 2012; Otis et al., 1950). The relationship between *Ẇ*_B_ and ${\dot{V}}_{E}$ is exponential (Molgat‐Seon et al., 2019), and the relative contributions of each of the main components of *Ẇ*_B_ vary depending on ${\dot{V}}_{E}$. When ${\dot{V}}_{E}$ is relatively low, such as at rest or during low‐intensity exercise, flow through the airways is predominantly laminar, and the viscoelastic component of *Ẇ*_B_ represents the largest portion of the total *Ẇ*_B_ (Otis et al., 1950). As ${\dot{V}}_{E}$ increases, flow becomes transitional and eventually turbulent, particularly in the large conducting airways (i.e., generations 0–4), leading to an exponential rise in *Ẇ*_B_ due to the marked increase in the resistive component of *Ẇ*_B_ (Dominelli & Sheel, 2012). Although this general pattern is evident in both males and females, sex has an important influence on the extent to which *Ẇ*_B_ increases as a function of ${\dot{V}}_{E}$ (Dominelli, Molgat‐Seon et al., 2015, 2015; Guenette et al., 2007; Mann et al., 2020; Molgat‐Seon et al., 2018; Peters et al., 2021; Wanke et al., 1991).

At rest and during low‐ to moderate‐intensity exercise, *Ẇ*_B_ is similar between males and females; however, when ${\dot{V}}_{E}$ is above ∼50–60 L min^−1^, *Ẇ*_B_ in females increases disproportionally relative to males (Dominelli, Molgat‐Seon et al., 2015, 2015; Guenette et al., 2007; Mann et al., 2020; Molgat‐Seon et al., 2018; Peters et al., 2021; Wanke et al., 1991). At fixed levels of absolute ${\dot{V}}_{E}$, flow is likely more laminar in males than in females due to sex differences in airway size (Dominelli et al., 2018), but the elasticity of the lungs and airways does not differ based on sex (Colebatch et al., 1979). Thus, the viscoelastic component of *Ẇ*_B_ is similar in males and females throughout exercise (Guenette et al., 2009; Molgat‐Seon et al., 2019; Peters et al., 2021). It follows that sex differences in *Ẇ*_B_ during exercise are due to females having a higher resistive component of *Ẇ*_B_ than males (Dominelli, Molgat‐Seon et al., 2015; Guenette et al., 2009; Mann et al., 2020; Molgat‐Seon et al., 2019; Peters et al., 2021), which is thought to be the result of sex differences in airway size. Several studies indicate that females have smaller large conducting airways than males (Bhatt et al., 2022; Christou et al., 2021; Dominelli et al., 2018; Martin et al., 1987; Molgat‐Seon et al., 2023; Peters et al., 2021; Sheel et al., 2009), which persists even when accounting for lung size or height (Christou et al., 2021; Dominelli et al., 2018; Molgat‐Seon et al., 2023; Sheel et al., 2009). Dysanapsis refers to the unequal growth trajectories of the lungs and airways (Mead, 1980), which can be quantified by calculating the quotient of a measure of airway size (e.g., airway luminal area) and a measure of lung volume (e.g., total lung capacity; TLC) (Molgat‐Seon et al., 2023; Smith et al., 2020). On average, females have a smaller airway‐to‐lung (i.e., dysanapsis) ratio than males (Molgat‐Seon et al., 2023; Smith et al., 2020, 2022), which suggests that females have proportionally narrower airways than their male counterparts. The geometric properties of the airways have a profound influence on airway resistance (Hogg et al., 2017), which affects the resistive component of *Ẇ*_B_. Thus, the sex differences in airway size are thought to be the underlying mechanism of sex differences in *Ẇ*_B_. However, males tend to have great lung volumes than females and all studies examining the effect of sex on *Ẇ*_B_ during exercise have compared males and females that differ in terms of forced vital capacity (FVC) and/or TLC (Dominelli, Molgat‐Seon et al., 2015, 2015; Guenette et al., 2007; Mann et al., 2020; Molgat‐Seon et al., 2018; Peters et al., 2021; Wanke et al., 1991).

Despite considerable variability (Vameghestahbanati et al., 2024), there is a significant association between TLC and airway size (Molgat‐Seon et al., 2023; Peters et al., 2021), which implies that, on average, individuals with a larger TLC tend to have larger airways than those with a smaller TLC. Given the aforementioned effect of airway size on the resistive component of *Ẇ*_B_ and that males tend to have a larger TLC and FVC than females (Hall et al., 2021; Quanjer et al., 2012), it could be argued that the effect of sex on *Ẇ*_B_ during exercise may be confounded by male–females differences in lung size, yet no previous studies have considered the effect of FVC or TLC on sex differences in *Ẇ*_B_. In order to confirm that sex does in fact have an independent effect on *Ẇ*_B_ during exercise, a prospective study that considers how *Ẇ*_B_ during exercise is affected by male–female differences in a measure of lung size (e.g., TLC or FVC) is required.

Accordingly, the aim of this study is to characterize the relationship between *Ẇ*_B_ and ${\dot{V}}_{E}$ during exercise in healthy males and females by accounting for sex differences in lung size. Although TLC is the most accurate measure of lung size, we used FVC as an index of lung size since it can be measured using spirometry, represents approximately 70–75% of TLC in healthy adults (Ruppel, 2012) and is closely correlated with TLC (Hoftman et al., 2017). We hypothesized that females have a higher *Ẇ*_B_ for a given ${\dot{V}}_{E}$ than males after accounting for sex differences in FVC.

## 2 METHODS

### 2.1 Ethical approval

All study procedures were approved by the University of Winnipeg Human Research Ethics Board (no. HE18219), which adheres to principles outlined in the Government of Canada's *Tri‐Council Policy Statement: Ethical Conduct for Research Involving Humans* and the *Declaration of Helsinki*, except for registration in a database.

### 2.2 Participants

After providing written informed consent, a multi‐ethnic sample of *n* = 30 healthy adults (*n* = 15 males and *n* = 15 females) participated in the study. The inclusion criteria were age between 20 and 60 years, body mass index ≥18 and ≤30 kg m^−2^ and normal spirometry based on predicted normal values (Quanjer et al., 2012). Participants were excluded if they were current smokers or had previously smoked ≥5 pack‐years, had a history of respiratory or cardiovascular disease, had a medical condition that would interfere with their ability to exercise or had any contraindications to the oesophageal balloon catheter (see Section 2.4.3). A subset of *n* = 7 males and *n* = 7 females that were matched based on FVC (mean difference: 0.21 ± 0.10 L) and age (mean difference: 1.4 ± 1.3 years) to compare *Ẇ*_B_ during exercise between males and females in the absence of sex differences in lung size.

### 2.3 Experimental overview

Participants reported to the laboratory on one occasion. Testing involved the measurement of participants' height and body mass, spirometry, and an incremental cycle exercise test to volitional exhaustion. Before the exercise test, participants were instrumented with an oesophageal balloon catheter (see Section 2.4.3). The exercise test was performed on a cycle ergometer (Ergoselect 200K, Ergoline GmbH, Bitz, Germany) and consisted of a 5–10 min rest period while participants were seated on the ergometer, followed by stepwise increases in work rate by 20 W every 2 min starting at 20 W until volitional exhaustion. During the last 30 s of rest and each exercise stage, the participants were asked to perform an inspiratory capacity (IC) manoeuvre. A maximal effort during the incremental cycle exercise test was confirmed based on one or more of the following criteria: (i) evidence of a plateau in oxygen uptake despite increasing work rate (i.e., <0.15 L min^−1^ increase in oxygen uptake between the final two exercise stages), (ii) respiratory exchange ratio ≥1.15, (iii) and heart rate ≥90% of predicted maximal heart rate (American Thoracic Society & American College of Chest Physicians, 2003; Tanaka et al., 2001).

### 2.4 Measurements

#### 2.4.1 Spirometry

Spirometry was performed according to standard recommendations (Graham et al., 2019) using a portable spirometer (Minispir, Medical International Research, Rome, Italy). Participants performed FVC manoeuvres in triplicate or until repeatability for FVC and forced expired volume in 1 s was met. Each participant's FVC, forced expired volume in 1 s (FEV_1_) and forced expiratory flow between 25% and 75% of FVC (FEF_25–75_) were expressed in absolute units and as a percentage of predicted normal values (Quanjer et al., 2012).

#### 2.4.2 Ventilatory, cardiovascular and metabolic response to exercise

At rest and throughout exercise, standard ventilatory, cardiovascular and metabolic variables were measured using a customized system composed of open‐circuit spirometry and expired gas analysis. Participants breathed through a mouthpiece and two‐way non‐rebreathe valve (Series 2700B, Hans Rudolph, Shawnee, KS, USA), connected to large‐bore tubing on the inspired and expired limbs. Inspired and expired airflow were measured using independent pneumotachometers (Series 4813, Hans Rudolph), which were calibrated using a 3 L syringe. The expired pneumotachometer was heated to 37°C while the inspired air was at ambient temperature. The fractions of mixed expired oxygen and carbon dioxide were sampled from a mixing chamber connected to the expiratory limb of the breathing circuit and measured using a gas analyser (14‐10000, CWE Inc., Ardmore, PA, USA). Arterial oxygen saturation was estimated using finger pulse oximetry (Model 7500, Nonin Medical Inc., Plymouth, MN, USA). Heart rate was measured using a telemetric sensor (T34, Polar, Kempele, Finland).

#### 2.4.3 Work of breathing

Oesophageal pressure (*P*_OES_) was measured using a nasogastric catheter (ESO101, Top Pine Technology Development Ltd, Hong Kong, China). Prior to inserting the catheter, a topical anaesthetic spray (Lidocan endotracheal spray; Odan Laboratories, Montreal, QC, Canada) was used to numb the skin inside the participant's nostril and throat. Placement of the catheter was performed as previously described (Zin & Milic‐Emili, 2005), with 0.5 mL of air placed in the oesophageal balloon. *P*_OES_ was measured using a differential pressure transducer (DP15‐34, Validyne Engineering, Northridge, CA, USA) and the validity of the *P*_OES_ signal was verified by performing the conventional occlusion test using an separate mouthpiece connected to a differential pressure transducer (DP15‐34, Validyne Engineering) (Baydur et al., 1982). For each participant at rest and each stage of exercise, a composite average *P*_OES_–volume curve was generated. The *P*_OES_–volume curve was then divided into three sections representing inspiratory resistive, inspiratory elastic and expiratory resistive components of *Ẇ*_B_ (i.e., the Hedstrand diagram) and the total *Ẇ*_B_ was calculated based on the sum of the areas of each section, as previously described (Cross et al., 2021; Hedstrand, 1969). Each participant's *Ẇ*_B_ data were then plotted as a function of absolute ${\dot{V}}_{E}$, and curves were fit to each individual participant's *Ẇ*_B_–${\dot{V}}_{E}$ data according to the following equation:

(1) $${\dot{W}}_{B}=a{{\dot{V}}_{E}}^{3}+b{{\dot{V}}_{E}}^{2}$$

where, for a given absolute ${\dot{V}}_{E}$, *a* ${\dot{V}}_{E}$ ^3^ represents the resistive component of *Ẇ*_B_ and *b* ${\dot{V}}_{E}$ ^2^ represents the viscoelastic component of *Ẇ*_B_. Although this model can be used to obtain estimates of the resistive and viscoelastic components of *Ẇ*_B_, as we and others have done previously (Guenette et al., 2007; Margaria et al., 1960; Molgat‐Seon et al., 2019), we used this analytical approach in the context of the present study to facilitate comparisons of the *Ẇ*_B_–${\dot{V}}_{E}$ relationship between the sexes. To do so, we determined the total *Ẇ*_B_ for each participant at discrete levels of absolute ${\dot{V}}_{E}$ by solving Equation 1 for each participant in successive 10 L min^−1^ increments from 10 L min^−1^ up to each participant's maximal ${\dot{V}}_{E}$. We also calculated the area under the *Ẇ*_B_–${\dot{V}}_{E}$ curve in order to assess the magnitude of the difference in *Ẇ*_B_ between the sexes across the entire sample and in the participants matched for FVC and age.

#### 2.4.4 Data sampling and analysis

At rest and during exercise, all raw data were sampled at 1000 Hz and recorded using a PowerLab 8/30 analog‐to‐digital converter (16/35, ADInstruments, Colorado Springs, CO, USA) running LabChart Pro software (V8.1.21, AD Instruments) for subsequent analysis. Approximately 30‐s segments of data from rest, the last minute of each exercise stage and peak exercise were analysed. Oxygen uptake, carbon dioxide production, respiratory exchange ratio, heart rate and arterial oxygen saturation were averaged. Flow, volume and *P*_OES_ data were processed using a custom program written in Python to generate average tidal flow–volume and pressure–volume curves and calculate IC, which were then used to calculate tidal volume, breathing frequency, ${\dot{V}}_{E}$, inspiratory and expiratory reserve volumes and *Ẇ*_B_.

#### 2.4.5 Statistical analysis

All data were tested for normality using the Shapiro–Wilk test; rank transformations were performed prior to analysis for variables that were not normally distributed (Conover & Iman, 1981). Descriptive characteristics and anthropometric data, spirometry data, peak exercise data and *Ẇ*_B_ at specific levels of ${\dot{V}}_{E}$ (i.e., ∼35 and ∼70 L min^−1^) were compared between the sexes using an independent samples Student's *t*‐test or the Mann–Whitney *U*‐test. Ventilatory parameters (i.e., tidal volume, breathing frequency, ${\dot{V}}_{E}$, as well as inspiratory and expiratory reserve volumes) were compared between the sexes at rest and standardized submaximal work rates (i.e., from 0 W to the highest equivalent submaximal work rate of 100 W) using a two‐way repeated measures analysis of variance. If significant *F*‐ratios were detected, a pairwise comparisons Tukey's *post hoc* test was used to determine the location of group mean differences. Across all participants, we determined the effect of ${\dot{V}}_{E}$, sex and their interaction on *Ẇ*_B_ by accounting for FVC using a repeated measures analysis of covariance. *Ẇ*_B_ was compared between the sexes at the following levels of ${\dot{V}}_{E}$: 10, 20, 30, 40, 50, 60, 70 and 80 L min^−1^. If significant *F*‐ratios were detected, a pairwise comparisons Tukey's *post hoc* test was used to determine the location of group mean differences. In the subset of males and females matched for FVC and age, we determined the effect of ${\dot{V}}_{E}$, sex and their interaction on *Ẇ*_B_ using a repeated measures analysis of variance. *Ẇ*_B_ was compared between the sexes at the following levels of ${\dot{V}}_{E}$: 10, 20, 30, 40, 50, 60, 70, 80 and 90 L min^−1^. If significant *F*‐ratios were detected, a pairwise comparisons Tukey's *post hoc* test was used to determine the location of group mean differences. Linear regression analysis was used to determine the relationship between FVC and *Ẇ*_B_ at absolute levels of ${\dot{V}}_{E}$ achieved by all participants (i.e., ∼35 L min^−1^ and ∼70 L min^−1^) across the entire sample and within each sex. All statistical analyses were performed using the open‐access statistical software package Jamovi (Jamovi v1.2, Sydney, Australia), with the level of significance set at *P *< 0.05. All data are reported as the mean ± standard deviation.

## 3 RESULTS

### 3.1 Participant characteristics and spirometry data

Participant characteristics and spirometry data are shown in Table 1. Across all participants, males and females had a similar age (*P *= 0.617) and body mass index (*P *= 0.308), but males had a significantly greater height (*P *= 0.042) and body mass (*P *= 0.037) than females. Additionally, males had significantly greater FVC (*P *= 0.001), FEV_1_ (*P *= 0.001) and FEF_25–75_ (*P *= 0.023) than females; however, FEV_1_/FVC was similar between sexes (*P *= 0.548). When values were expressed as a percentage of predicted normal values, FVC (*P *= 0.542), FEV_1_ (*P *= 0.169) and FEF_25–75_ (*P *= 0.499) were not significantly different between the sexes. In the subset of age‐ and FVC‐matched males and females, there were no sex differences in age (*P *= 0.606), height (*P *= 0.670), body mass (*P *= 0.912) and body mass index (*P *= 0.945). Moreover, there were no significant differences in FVC (*P *= 0.671), %‐predicted FVC (*P *= 0.062), FEV_1_ (*P *= 0.413), %‐predicted FEV_1_ (*P *= 0.110), FEV_1_/FVC (*P *= 0.467), FEF_25–75_ (*P *= 0.676) or %‐predicted FEF_25–75_ (*P *= 0.207) between the sexes.

**TABLE 1:** Physical characteristics and spirometry data.

	Males	Females	*P*
All participants	*n* = 15	*n* = 15	
Physical characteristics			
Age, years	29 ± 11	29 ± 13	0.617
Height, cm	176 ± 7	170 ± 8	0.042^*^
Body mass, kg	78 ± 11	69 ± 11	0.037^*^
Body mass index, kg m^−2^	25 ± 2	24 ± 4	0.308
Spirometry			
FVC, L	5.59 ± 1.03	4.15 ± 0.78	<0.001^*^
FVC, % predicted	106 ± 14	103 ± 12	0.542
FEV_1_, L	4.59 ± 0.89	3.33 ± 0.59	<0.001†
FEV_1_, % predicted	105 ± 15	98 ± 11	0.169
FEV_1_/FVC	83 ± 5	81 ± 10	0.495
FEF_25–75_, L sec^−1^	4.58 ± 1.23	3.49 ± 1.26	0.023^*^
FEF_25–75_, % predicted	104 ± 22	97 ± 30	0.499
Participants matched for age and FVC	*n* = 7	*n* = 7	
Physical characteristics			
Age, years	33 ± 15	32 ± 15	0.606
Height, cm	172 ± 7	174 ± 9	0.670
Body mass, kg	73 ± 12	73 ± 11	0.912
Body mass index, kg m^−2^	25 ± 3	24 ± 4	1.000
Spirometry			
FVC, L	4.89 ± 0.60	4.73 ± 0.71	0.671
FVC, % predicted	101 ± 10	111 ± 8	0.062
FEV_1_, L	4.00 ± 0.40	3.81 ± 0.43	0.413
FEV_1_, % predicted	100 ± 11	108 ± 4	0.110
FEV_1_/FVC	84 ± 6	81 ± 8	0.467
FEF_25–75_, L sec^−1^	3.94 ± 0.62	3.76 ± 0.90	0.676
FEF_25–75_, % predicted	97 ± 18	108 ± 10	0.207

*Note*: Data were compared between the sexes using independent samples *t*‐tests or the Mann‐Whitney *U*‐test. *Significant difference between the sexes (*P *< 0.05). Abbreviations: FEF_25–75_, forced expired flow between 25 and 75% of FVC; FEV_1_, forced expired volume in 1 s; FVC, forced vital capacity.

### 3.2 Cardiorespiratory responses to exercise

**FIGURE 1:**
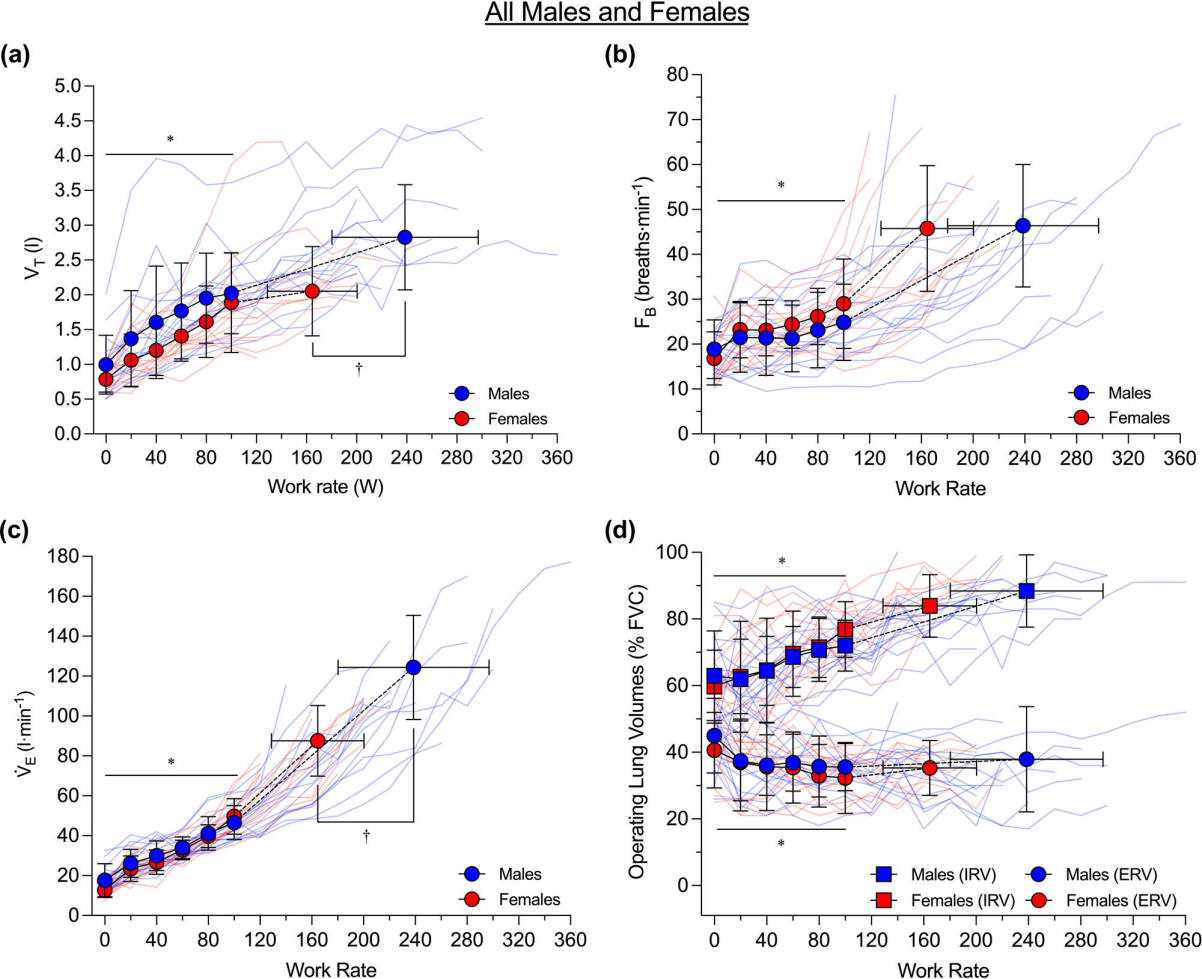
Ventilatory response to incremental cycle exercise in all males (*n* = 15) and females (*n* = 15). The highest equivalent submaximal work rate achieved by all subjects was 100 W. Dashed lines within each group connect the 100 W data point to the peak exercise data point. Group average data are shown as blue circles for males and red circles for females, and raw data for each participant are shown as light blue lines for males and light red lines for females. In (d), which depicts operating lung volumes, inspiratory reserve volume data are shown as squares instead of circles for both males and females, and expiratory reserve volume data are shown as circles. Data were compared between the sexes at rest and standardized submaximal work rates using two‐way repeated measures analyses of variance and at peak using independent sample *t*‐tests (see Section 2.4.5 for additional information). *Significant effect of work rate (*P *< 0.05); †significant difference between the sexes (*P *< 0.05). ERV, expiratory reserve volume; *F*_B_, breathing frequency; FVC, forced vital capacity; IRV, inspiratory reserve volume; ${\dot{V}}_{E}$, minute ventilation; *V*_T_, tidal volume.

**FIGURE 2:**
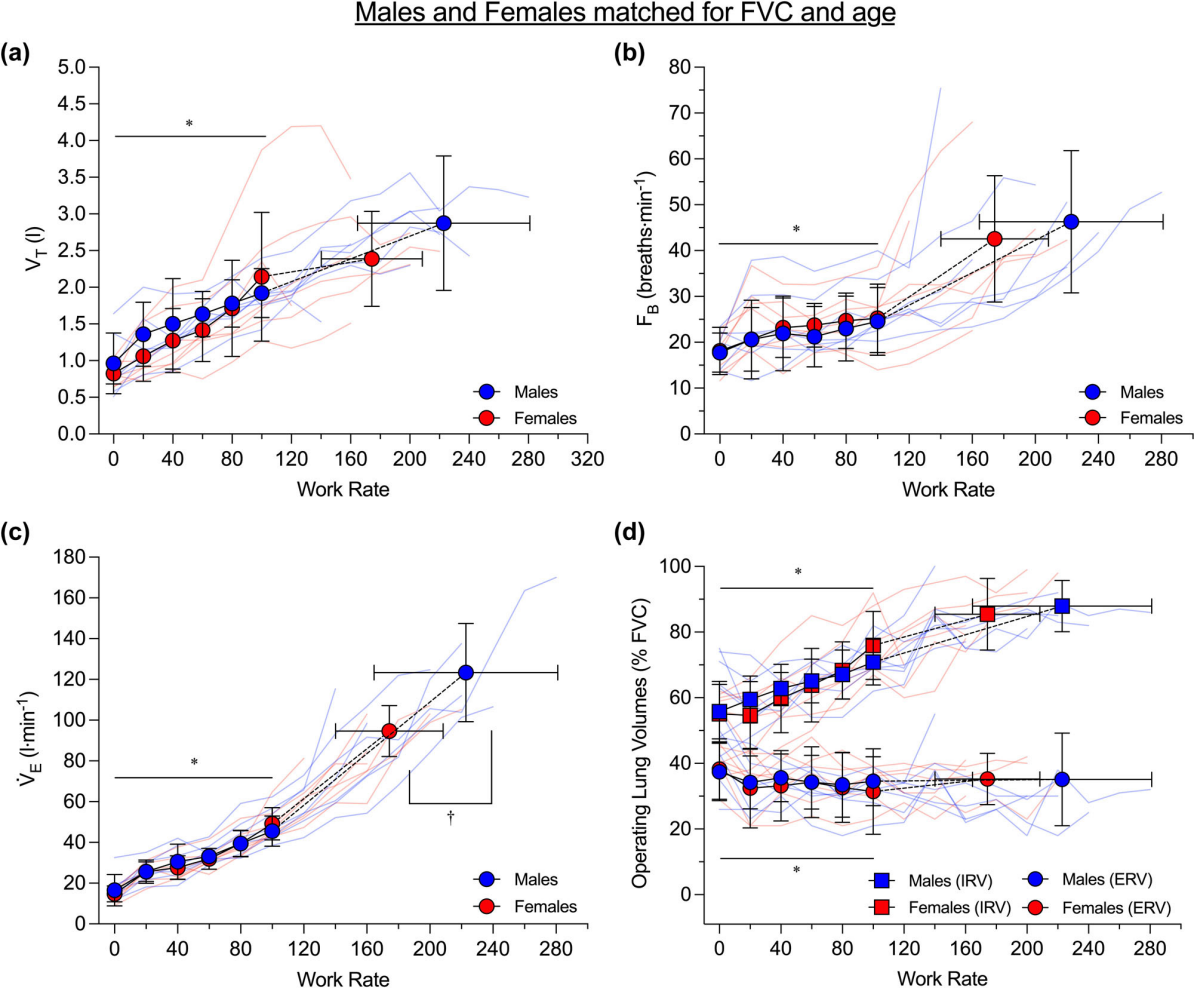
Ventilatory response to incremental cycle exercise in the subset of males (*n* = 7) and females (*n* = 7) who were matched for FVC and age. The highest equivalent submaximal work rate achieved by all subjects was 100 W. Dashed lines within each group connect the 100 W data point to the peak exercise data point. Group average data are shown as blue circles for males and red circles for females, and raw data for each participant are shown as light blue lines for males and light red lines for females. In (d), which depicts operating lung volumes, inspiratory reserve volume data are shown as squares instead of circles for both males and females, and expiratory reserve volume data are shown as circles. Data were compared between the sexes at rest and standardized submaximal work rates using two‐way repeated measures analyses of variance and at peak using independent sample *t*‐tests (see Section 2.4.5 for additional information). *Significant effect of work rate (*P *< 0.05); †significant difference between the sexes (*P *< 0.05). ERV, expiratory reserve volume; *F*_B_, breathing frequency; FVC, forced vital capacity; IRV, inspiratory reserve volume; ${\dot{V}}_{E}$, minute ventilation; *V*_T_, tidal volume.

Ventilatory responses to exercise are shown in Figure 1 for all participants and in Figure 2 for the subset of males and females matched for FVC and age. A summary of the results of statistical analyses of the effects of work rate, sex and their interaction on ventilatory parameters during submaximal exercise is provided in Table 2. From rest up to the highest equivalent submaximal work rate achieved by all participants (i.e., 100 W) there was a significant main effect of work rate but not sex or the interaction between work rate and sex on tidal volume, breathing frequency, ${\dot{V}}_{E}$, expiratory reserve volume and inspiratory reserve volume during exercise across all participants and in the subset of males and females matched for FVC and age. At peak exercise, males had a significantly higher tidal volume (*P *= 0.003) and ${\dot{V}}_{E}$ (*P *= 0.001) than females, but a similar breathing frequency (*P *= 0.757), expiratory reserve volume (*P *= 0.653) and inspiratory reserve volume (*P *= 0.181) across all participants. In the subset of males and females matched for FVC and age, males had a significantly higher ${\dot{V}}_{E}$ than females at peak exercise than females (*P *= 0.016), but tidal volume (*P *= 0.617), breathing frequency (*P *= 0.311), expiratory reserve volume (*P *= 0.259) and inspiratory reserve volume (*P *= 0.889) were similar between the sexes.

**TABLE 2:** Summary of the results of statistical analyses of the effects of work rate, sex and their interaction on ventilatory parameters during submaximal exercise.

	Main effects		Interaction effect
	Work rate	Sex	Work rate × Sex
All participants (*n* = 15 males, *n* = 15 females)			
Tidal volume, L	<0.0001^*^	0.119	0.703
Breathing frequency, breaths min^−1^	<0.0001^*^	0.560	0.054
${\dot{V}}_{E}$, L min^−1^	<0.0001^*^	0.206	0.151
Expiratory reserve volume, % FVC	<0.0001^*^	0.642	0.685
Inspiratory reserve volume, % FVC	<0.0001^*^	0.645	0.530
Participants matched for age and FVC (*n* = 7 males, *n* = 7 females)			
Tidal volume, L	<0.0001^*^	0.871	0.173
Breathing frequency, breaths min^−1^	<0.0001^*^	0.763	0.226
${\dot{V}}_{E}$	<0.0001^*^	0.333	0.828
Expiratory reserve volume, % FVC	<0.0001^*^	0.818	0.352
Inspiratory reserve volume, % FVC	<0.0001^*^	0.771	0.183

* Significant main effect of work rate (*P *< 0.05). Abbreviations: FVC, forced vital capacity; ${\dot{V}}_{E}$, minute ventilation.

Metabolic and cardiovascular responses at peak exercise for all participants and for the subset of males and females matched for FVC and age are shown in Table 3. Across all participants, males had a significantly higher work rate (*P *= 0.467), absolute oxygen uptake (i.e., in L min^−1^) (*P *= 0.001), relative oxygen uptake (i.e., in mL kg^−1^ min^−1^) (*P *= 0.001) and carbon dioxide output (*P *= 0.001) at peak exercise were higher in males than in females, whereas %‐predicted maximal oxygen uptake (*P *= 0.402), respiratory exchange ratio (*P *= 0.984), the ventilatory equivalents for oxygen (*P *= 0.412) and carbon dioxide (*P *= 0.301), heart rate (*P *= 0.585), and %‐predicted maximal heart rate (*P *= 0.787) were not different between the sexes. In the subset of males and females matched for FVC and age, work rate (*P *= 0.146), absolute oxygen uptake (*P *= 0.058), %‐predicted maximal oxygen uptake (*P *= 0.716), respiratory exchange ratio (*P *= 0.446), the ventilatory equivalents for oxygen (*P *= 0.970) and carbon dioxide (*P *= 0.722), heart rate (*P *= 0.875), and %‐predicted maximal heart rate (*P *= 0.716) at peak exercise were not different between the sexes, but males had a significantly higher relative oxygen uptake (*P *= 0.017) and carbon dioxide output (*P *= 0.030) than females.

**TABLE 3:** Metabolic and cardiovascular data at peak exercise.

	Males	Females	*P*
All participants	*n* = 15	*n* = 15	
Work rate, W	239 ± 58	163 ± 38	<0.001^*^
${\dot{V}}_{O_{2}}$, L min^−1^	3.58 ± 0.77	2.28 ± 0.55	<0.001^*^
${\dot{V}}_{O_{2}}$, mL kg^−1^ min^−1^	46 ± 8	33 ± 7	<0.001^*^
${\dot{V}}_{O_{2}}$, % predicted	113 ± 20	104 ± 35	0.402
${\dot{V}}_{CO_{2}}$, L min^−1^	4.12 ± 0.81	2.64 ± 0.59	<0.001^*^
RER	1.16 ± 0.10	1.16 ± 0.08	0.984
${\dot{V}}_{E}$/${\dot{V}}_{O_{2}}$	35 ± 7	38 ± 7	0.412
${\dot{V}}_{E}$/${\dot{V}}_{CO_{2}}$	30 ± 4	32 ± 6	0.301
HR, beats min^−1^	182 ± 12	179 ± 11	0.585
HR, % predicted	97 ± 5	96 ± 6	0.787
Participants matched for age and FVC	*n* = 7	*n* = 7	
Work rate, W	209 ± 47	174 ± 34	0.146
${\dot{V}}_{O_{2}}$, L min^−1^	3.09 ± 0.95	2.55 ± 0.48	0.058
${\dot{V}}_{O_{2}}$, mL kg^−1^ min^−1^	46 ± 8	36 ± 7	0.017^*^
${\dot{V}}_{O_{2}}$, % predicted	116 ± 28	110 ± 36	0.716
${\dot{V}}_{CO_{2}}$, L min^−1^	3.85 ± 1.10	2.91 ± 0.55	0.030^*^
RER	1.13 ± 0.06	1.14 ± 0.06	0.446
${\dot{V}}_{E}$/${\dot{V}}_{O_{2}}$	38 ± 8	38 ± 6	1.000
${\dot{V}}_{E}$/${\dot{V}}_{CO_{2}}$	33 ± 7	33 ± 5	0.722
HR, beats min^−1^	182 ± 14	181 ± 9	0.875
HR, % predicted	99 ± 4	97 ± 6	0.716

* Significant difference between the sexes (*P *< 0.05). Abbreviations: HR, heart rate; RER, respiratory exchange ratio; ${\dot{V}}_{CO_{2}}$, ${\dot{V}}_{E}$/${\dot{V}}_{CO_{2}}$_,_ ventilatory equivalent for carbon dioxide; ${\dot{V}}_{E}$/${\dot{V}}_{O_{2}}$_,_ ventilatory equivalent for oxygen; ${\dot{V}}_{O_{2}}$, oxygen uptake.

### 3.3 Work of breathing

**FIGURE 3:**
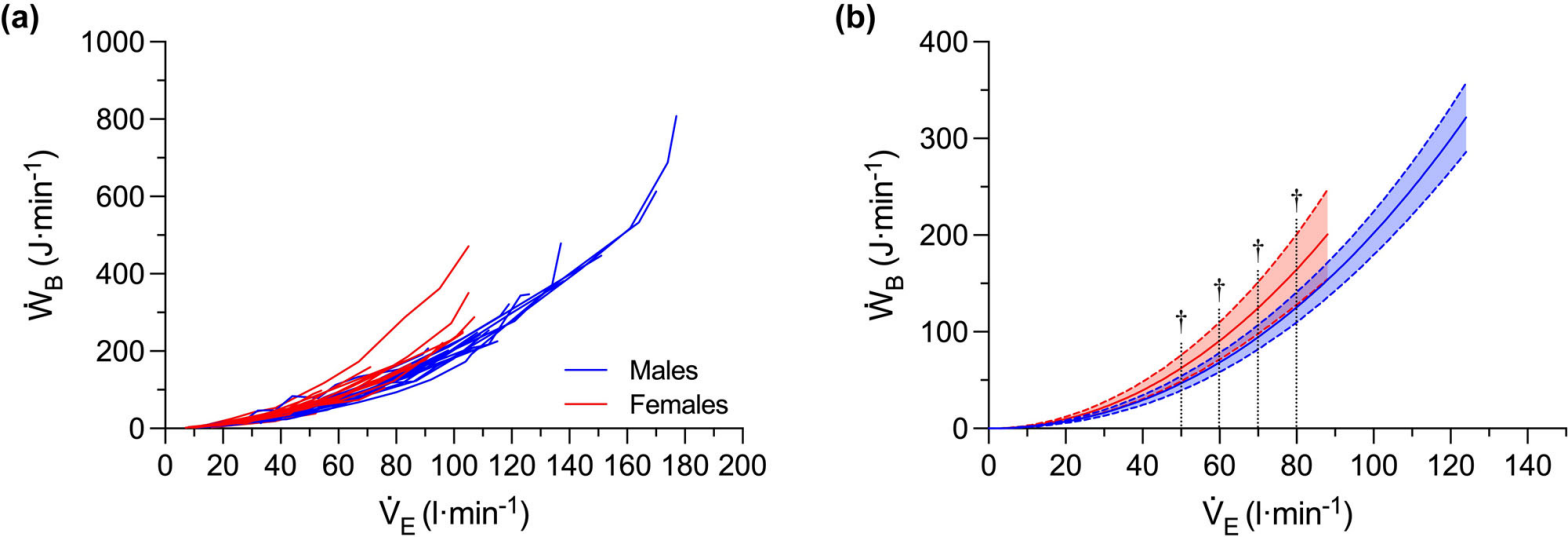
The relationship between *Ẇ*_B_ and minute ventilation during incremental cycle exercise across all males (*n* = 15) and females (*n* = 15). Individual data are shown in (a), and group‐mean curves are shown in (b). All mean curves are based on mean values from Equation 1, and each curve has been extrapolated to the average peak minute ventilation within each group. A repeated measures analysis of covariance was used to compare *Ẇ*_B_ between the sexes by accounting for FVC at the following levels of minute ventilation: 10, 20, 30, 40, 50, 60, 70, 80 and 90 L min^−1^ (see Section 2.4.5 for additional information). †Significant difference between the sexes (*P *< 0.05). ${\dot{V}}_{E}$, minute ventilation; *Ẇ*_B_, work of breathing.

**FIGURE 4:**
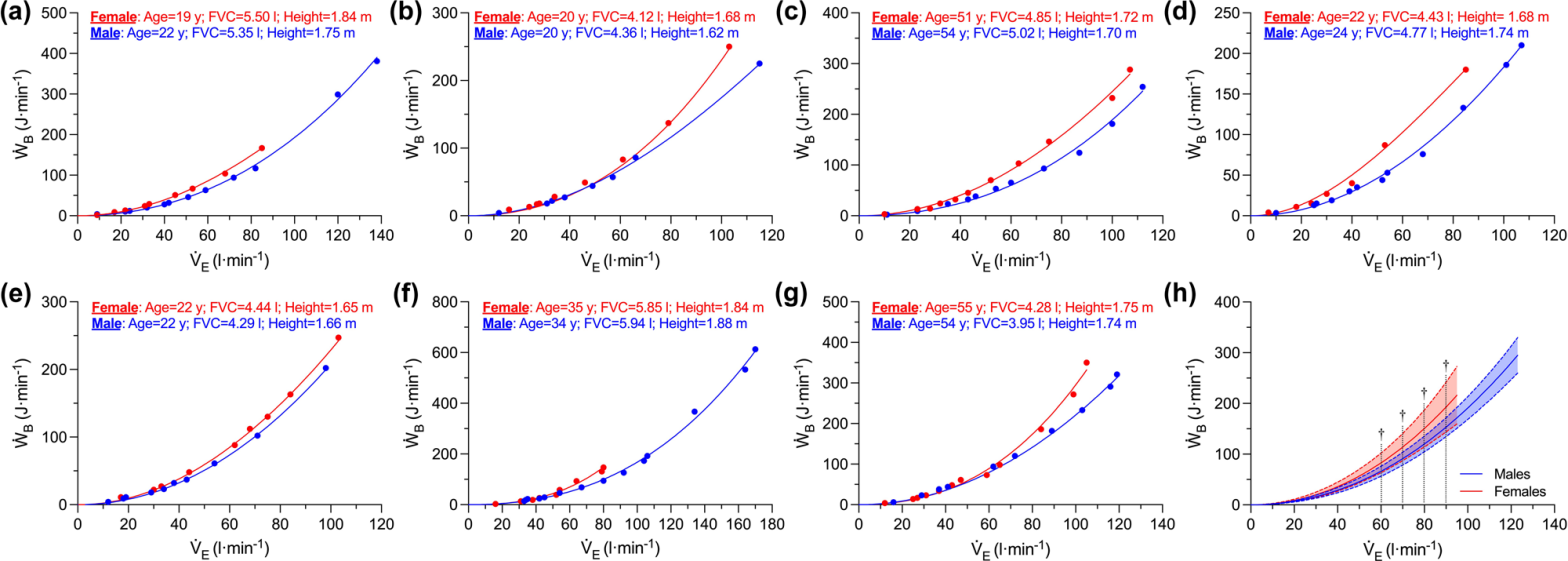
The relationship between *Ẇ*_B_ and minute ventilation during incremental cycle exercise in the subset of males (*n* = 7) and females (*n* = 7) matched for FVC and age. (a–g) Pairs of males and females; the *Ẇ*_B_–minute ventilation curve is based on values from Equation 1, with each individual's curve extrapolated to the highest minute ventilation they achieved. (h) Group‐mean *Ẇ*_B_–minute ventilation curves for the matched males and females, with each curve extrapolated to the average peak minute ventilation within each group. A two‐way repeated measures analysis of variance was used to compare *Ẇ*_B_ between the sexes at the following levels of minute ventilation: 10, 20, 30, 40, 50, 60, 70, 80 and 90 L min^−1^ (see Section 2.4.5 for additional information). †Significant difference between the sexes (*P *< 0.05). ${\dot{V}}_{E}$, minute ventilation; *Ẇ*_B_, work of breathing.

The relationship between *Ẇ*_B_ and ${\dot{V}}_{E}$ is shown in Figure 3 for all males and females and Figure 4 for the subset of males and females matched for age and FVC. Each participant's *Ẇ*_B_–${\dot{V}}_{E}$ data were fit to Equation 1, and the overall fit was excellent for all participants (mean *r* ^2^: 0.995 ± 0.006; range: 0.978–0.999; all *P *< 0.0001). Across all males and females, there was a main effect of ${\dot{V}}_{E}$ (*P *= 0.001) and sex (*P *= 0.022), as well as the interaction between ${\dot{V}}_{E}$ and sex on *Ẇ*_B_ during exercise (*P *= 0.001). FVC was included in the model as a covariate but was not significant (*P *= 0.323). Females had a significantly higher *Ẇ*_B_ than males at a ${\dot{V}}_{E}$ of 50 (*P *= 0.030), 60 (*P *= 0.023), 70 (*P *= 0.021) and 80 L min^−1^ (*P *= 0.020) (Figure 3b). A similar finding was observed in the subset of males and females matched for FVC and age, whereby there was a main effect of ${\dot{V}}_{E}$ (*P *= 0.001) and sex (*P *= 0.001), as well as the interaction between ${\dot{V}}_{E}$ and sex on *Ẇ*_B_ during exercise (*P *= 0.001). Within this subset of participants, females have a higher *Ẇ*_B_ than males at a ${\dot{V}}_{E}$ of 60 (*P *= 0.049), 70 (*P *= 0.019), 80 (*P *= 0.020) and 90 L min^−1^ (*P *= 0.014) (Figure 4h). Across all participants, the area under the *Ẇ*_B_–${\dot{V}}_{E}$ curve up to a ${\dot{V}}_{E}$ of 87 L min^−1^ (i.e., the lowest group average peak ${\dot{V}}_{E}$) was significantly higher in females than in males (i.e., 5277 ± 1128 vs. 3984 ± 561 J min^−1^ L^−1^ min^−1^; *P = *0.001), which corresponds to a 32% difference between the sexes. In the males and females matched for FVC and age, the area under the *Ẇ*_B_–${\dot{V}}_{E}$ curve up to a ${\dot{V}}_{E}$ of 94 L min^−1^ (i.e., the lowest group average peak ${\dot{V}}_{E}$) was significantly higher in females than in males (i.e., 4340 ± 568 vs. 3850 ± 500 J min^−1^ L^−1^ min^−1^; *P *= 0.002), which corresponds to a 28% difference between the sexes.

**FIGURE 5:**
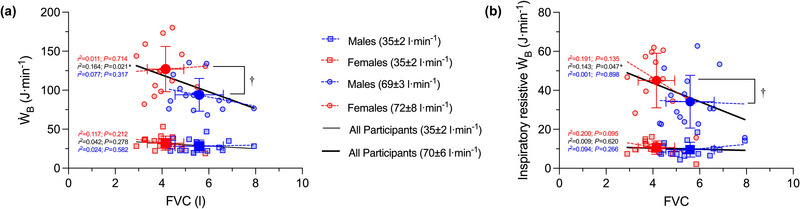
The relationship between *Ẇ*_B_ and FVC (a) and the inspiratory resistive *Ẇ*_B_ and FVC (b) at a ${\dot{V}}_{E}$ of 35 ± 2 and 70 ± 5 L min^−1^ across all males (*n* = 15) and females (*n* = 15). Mean and raw data are shown in red for females and blue for males. Dashed lines represent the association between *Ẇ*_B_ and FVC within each sex and level of minute ventilation, and the continuous black lines represent the association between the *Ẇ*_B_ and FVC across all participants at each level of minute ventilation. Linear regression analysis was used to determine the relationship between FVC and *Ẇ*_B_ at minute ventilations of ∼35 and ∼70 L min^−1^ across the entire sample and within each sex, and independent sample *t*‐tests were used to compare *Ẇ*_B_ and the inspiratory resistive component of *Ẇ*_B_ between the sexes at minute ventilations of ∼35 and ∼70 L min^−1^ (see Section 2.4.5 for additional information). †Significant difference between the sexes (*P *< 0.05). FVC, forced vital capacity; ${\dot{V}}_{E}$, minute ventilation; *Ẇ*_B_, work of breathing.

The relationships between *Ẇ*_B_ and FVC at a ${\dot{V}}_{E}$ of 35 ± 2 and 70 ± 6 L min^−1^ across all participants and within each sex are shown in Figure 5. There were no significant associations between FVC and *Ẇ*_B_ at a ${\dot{V}}_{E}$ of 35 ± 2 L min^−1^ across all participants (*r* ^2 ^= 0.042, *P *= 0.278), nor in males (*r* ^2 ^= 0.024, *P *= 0.582) and in females (*r* ^2 ^= 0.117, *P *= 0.312) separately. Likewise, there was no significant sex difference in *Ẇ*_B_ at a ${\dot{V}}_{E}$ of 35 ± 2 L min^−1^ (*P *= 0.135). There was a significant association between FVC and *Ẇ*_B_ at a ${\dot{V}}_{E}$ of 70 ± 5 L min^−1^ across all participants (*r* ^2 ^= 0.164; all *P *= 0.026); however, the association between FVC and *Ẇ*_B_ at a ${\dot{V}}_{E}$ of 70 ± 5 L min^−1^ was not significant within males (*r* ^2 ^= 0.077, *P *= 0.317) or females (*r* ^2 ^= 0.011, *P *= 0.714). *Ẇ*_B_ at a ${\dot{V}}_{E}$ of 70 ± 5 L min^−1^ was significantly higher in females than in males (*P *= 0.001). There were no significant associations between FVC and the inspiratory resistive *Ẇ*_B_ at a ${\dot{V}}_{E}$ of 35 ± 2 L min^−1^ across all participants (*r* ^2 ^= 0.009, *P *= 0.620), nor in males (*r* ^2 ^= 0.200, *P *= 0.095) and in females (*r* ^2 ^= 0.094, *P *= 0.266) separately. Likewise, there was no significant sex difference in the inspiratory resistive *Ẇ*_B_ at a ${\dot{V}}_{E}$ of 35 ± 2 L min^−1^ (*P *= 0.431). There was a significant association between FVC and the inspiratory resistive *Ẇ*_B_ at a ${\dot{V}}_{E}$ of 70 ± 5 L min^−1^ across all participants (*r* ^2 ^= 0.143; all *P *= 0.047); however, the association between FVC and the inspiratory resistive *Ẇ*_B_ at a ${\dot{V}}_{E}$ of 70 ± 5 L min^−1^ was not significant within males (*r* ^2 ^= 0.191, *P *= 0.135) or females (*r* ^2 ^= 0.001, *P *= 0.898). The inspiratory resistive *Ẇ*_B_ at a ${\dot{V}}_{E}$ of 70 ± 5 L min^−1^ was significantly higher in females than in males (*P *= 0.047).

## 4 DISCUSSION

### 4.1 Main findings

We examined the relationship between *Ẇ*_B_ and ${\dot{V}}_{E}$ during exercise in healthy males and females to determine the influence of FVC on sex differences in *Ẇ*_B_. Our main findings were three‐fold. First, after accounting for the effect of FVC, we noted that females had a significantly higher *Ẇ*_B_ than males at levels of ${\dot{V}}_{E}$ ranging from 50 to 80 L min^−1^. Second, we found that across all participants, FVC was not associated with the total *Ẇ*_B_ or the inspiratory resistive component of *Ẇ*_B_ during exercise eliciting a ${\dot{V}}_{E}$ of 35 ± 2 L min^−1^ and that although FVC was significantly associated with the total *Ẇ*_B_ and the inspiratory resistive component of *Ẇ*_B_ at a ${\dot{V}}_{E}$ of 70 ± 5 L min^−1^, this relationship was not evident within each sex. Third, we noted that in a subset of males and females matched for FVC and age, sex differences in the *Ẇ*_B_–${\dot{V}}_{E}$ relationship persisted, whereby females had a significantly higher *Ẇ*_B_ than males at levels of ${\dot{V}}_{E}$ ranging from 60 to 90 L min^−1^. Overall, our findings suggest that sex differences in *Ẇ*_B_ during exercise are independent of FVC, which further supports the notion that male–female differences in airway size are responsible for the higher resistive and total *Ẇ*_B_ during exercise in females relative to males.

### 4.2 Effects of sex and FVC on the work of breathing during exercise

The relationship between *Ẇ*_B_ and ${\dot{V}}_{E}$ has been well‐characterized in humans, ranging from healthy adults across the lifespan (Dominelli, Molgat‐Seon et al., 2015, 2015; Mann et al., 2020; Molgat‐Seon et al., 2018; Peters et al., 2021; Smith et al., 2018; Weavil et al., 2022), to younger and older endurance‐trained athletes (Guenette et al., 2007; Johnson et al., 1991, 1992), to patients with respiratory and cardiovascular disease (Cross et al., 2012; Guenette et al., 2014). During spontaneous breathing, *Ẇ*_B_ increases in a curvilinear manner as a function of ${\dot{V}}_{E}$. Overall, our data are closely aligned to those of previous studies in healthy males and females (Dominelli, Molgat‐Seon et al., 2015, 2015; Guenette et al., 2007; Mann et al., 2020; Molgat‐Seon et al., 2018; Peters et al., 2021; Wanke et al., 1991) (Figure 3). It is noteworthy that even in healthy adults, there is considerable variability in *Ẇ*_B_ for a given ${\dot{V}}_{E}$. For example, at a ${\dot{V}}_{E}$ of 70 ± 5 L min^−1^, *Ẇ*_B_ ranged from 68 to 180 J min^−1^ (Figure 5). The relationship between *Ẇ*_B_ and ${\dot{V}}_{E}$ is influenced by several factors, including the structural and mechanical properties of the lungs, airways and rib cage (Otis et al., 1950). Given that the morphology of the respiratory system is influenced by sex, it is logical that sex would have a corresponding impact on the *Ẇ*_B_–${\dot{V}}_{E}$ relationship. Indeed, several studies indicate that during exercise eliciting levels of ${\dot{V}}_{E}$ in excess of ∼50–60 L min^−1^, females have a higher *Ẇ*_B_ relative to their male counterparts (Dominelli, Molgat‐Seon et al., 2015, 2015; Guenette et al., 2007; Mann et al., 2020; Molgat‐Seon et al., 2018; Peters et al., 2021; Wanke et al., 1991). This is consistent with our data, whereby at levels of ${\dot{V}}_{E}$ ranging from 50 to 80 L min^−1^, *Ẇ*_B_ was significantly higher in females than in males (Figure 3). Although the available evidence suggests that sex differences in the *Ẇ*_B_ during exercise are due to sex differences in airway size, a caveat of previous work is that *Ẇ*_B_ is typically compared between the sexes across groups of males and females that differ in terms of measures of absolute lung size (i.e., TLC and/or FVC) (Dominelli & Molgat‐Seon, 2022). In theory, absolute lung size influences indices of mechanical ventilatory constraint at a given absolute ${\dot{V}}_{E}$, including breathing pattern, operating lung volumes and *Ẇ*_B_. It has been proposed that during exercise, relatively smaller lungs in females predispose them to relative hyperinflation, an increased reliance on breathing frequency and a greater encroachment on available ventilatory reserve, which would increase *Ẇ*_B_ for a given ${\dot{V}}_{E}$ (McClaran et al., 1998). Thus, it could be argued that the effect of sex on *Ẇ*_B_ during exercise may be driven or at least influenced by male–female differences in lung size; however, experimental data supporting this notion are lacking.

Although sex differences in airway luminal area are evident after controlling for TLC, comparisons of *Ẇ*_B_ in males and females that consider the potential influence of lung size are currently lacking. Thus, to account for the effect of lung size, effectively isolating the influence of sex differences in airway size on *Ẇ*_B_, we used three approaches. First, we compared the *Ẇ*_B_–${\dot{V}}_{E}$ relationship in a sample of males and females by accounting for the effect of FVC statistically. We found that FVC had no significant effect on the *Ẇ*_B_–${\dot{V}}_{E}$ relationship and that females had a significantly higher *Ẇ*_B_ than males at levels of ${\dot{V}}_{E}$ ranging from 50 to 80 L min^−1^ (Figure 3). Second, we compared the *Ẇ*_B_–${\dot{V}}_{E}$ relationship in a subset of males and females matched for FVC and age. We noted that although FVC was similar between the sexes, females had a higher *Ẇ*_B_ at levels of ${\dot{V}}_{E}$ ranging from 60 to 90 L min^−1^ (Figure 4). We also found that the magnitude of the sex difference in the *Ẇ*_B_–${\dot{V}}_{E}$ relationship was similar in the subset of males and females matched for FVC and age relative to all males and females (i.e., 32% vs. 28%). Third, we determined the association between FVC and both the total and the inspiratory resistive component of *Ẇ*_B_ at discrete levels of ${\dot{V}}_{E}$ across all participants and within each sex separately (Figure 5). We noted that FVC was not significantly associated with the total *Ẇ*_B_ or the inspiratory resistive component of *Ẇ*_B_ at a ${\dot{V}}_{E}$ of 35 ± 2 L min^−1^. Conversely, at a ${\dot{V}}_{E}$ of 70 ± 5 L min^−1^, there was a significant association between FVC and the total and inspiratory resistive component of *Ẇ*_B_ across all participants, but this relationship was no longer significant within each sex (i.e., Simpson's paradox). Overall, our results support the notion that sex differences in *Ẇ*_B_ during exercise are independent of FVC.

It is also noteworthy that in our sample, the ventilatory response to exercise was similar between the sexes (Figures 1 and 2). This observation differs from other studies, indicating that although males and females have a similar ${\dot{V}}_{E}$ for a given submaximal work rate, males have a higher tidal volume and a lower breathing frequency than females (Cory et al., 2015; Deruelle et al., 2008). The males in our study were of average stature, but the females were taller than average relative to the Canadian population (Statistics Canada, 2020). Thus, the lack of sex differences in most of the ventilatory parameters during submaximal exercise is likely due to the substantial overlap in height and FVC between the sexes. In the context of the present study, the fact that the ventilatory response to exercise was similar between the sexes facilitates sex‐based comparison of *Ẇ*_B_ during exercise, particularly in the subset of participants matched for age and FVC.

### 4.3 Mechanisms of sex differences in the work of breathing during exercise

Several studies have demonstrated that, on average, females have smaller airways than males even when matched for height or TLC (Christou et al., 2021; Dominelli et al., 2018; Molgat‐Seon et al., 2023; Sheel et al., 2009). In theory, sex differences in airway morphology influence *Ẇ*_B_ due to the profound influence of airway radius on the resistance to both laminar and turbulent flow (Hogg et al., 2017), which affects the resistive component of *Ẇ*_B_. Though physiologically sound, the evidence supporting this theory comes from cross‐sectional studies in males and females that involved the measurement of *Ẇ*_B_ but not airway size or the measurement of airway size but not *Ẇ*_B_. Recently, measures of *Ẇ*_B_ and fourth to eighth generation airway cross‐sectional area were obtained in healthy young males and females, and there was a significant association between airway cross‐sectional area and the resistive component of *Ẇ*_B_ (Peters et al., 2021). Moreover, when females breathe a normoxic helium–oxygen gas mixture during incremental exercise, an intervention that is physiologically analogous to acutely increasing airway luminal area, the resistive and total *Ẇ*_B_ is reduced to levels that are similar to those of their male counterparts breathing atmospheric air (Mann et al., 2020). Overall, our findings, as well as the available experimental evidence, provide convincing support for the notion that sex differences in *Ẇ*_B_ are due to male–female differences in airway morphology and not sex differences in lung size.

### 4.4 Limitations

Although our study provides insight regarding the influence of FVC on sex differences in *Ẇ*_B_ during exercise, there are important limitations that merit discussion. First, males typically have greater FVC and TLC than females (Hall et al., 2021; Quanjer et al., 2012), and regardless of sex, *Ẇ*_B_ for a given absolute ${\dot{V}}_{E}$ increases with age (Molgat‐Seon et al., 2018). It follows that accounting for the effect of FVC on sex differences in *Ẇ*_B_ requires that we match females and males for both FVC and age. Thus, the sample size of participants who could be matched for both FVC and age was relatively small. To overcome this limitation, we also opted to account for the effect of FVC statistically, using analysis of covariance and linear regression, to examine the effects of sex and FVC on the *Ẇ*_B_–${\dot{V}}_{E}$ relationship across all participants. In our view, this provides additional support for our findings in the subset of males and females matched for FVC. Second, we used FVC as an index of lung size instead of TLC due to the inability to measure residual volume. Given that all of our participants had normal spirometry, we believe that it is unlikely that slight differences in residual volume between the sexes would explain our findings. Third, because one aspect of this study required matching males and females for FVC, this analysis resulted in a selection of smaller‐than‐average males and larger‐than‐average females, meaning this subset of our sample may not be entirely representative of the general population. As previously noted, to circumvent this issue, we also determined the relationship between *Ẇ*_B_ and ${\dot{V}}_{E}$ across all males and females and accounted for the effect of FVC statistically.

### 4.5 Perspectives

Appropriately comparing the physiological responses to exercise between the sexes presents a problem related to allometric scaling. On average, important biological attributes that impact the physiological response to exercise, such as body mass and composition, height and lung size, differ between the sexes (Hall et al., 2021; Statistics Canada, 2020). It can be difficult to determine whether to compare outcome variables of interest between males and females in absolute terms or by normalizing to a given anthropometric measure. As we have previously argued (Dominelli & Molgat‐Seon, 2022), both approaches have merit and the chosen method of comparing the physiological responses to exercise in males and females depends on the experimental context. Since the initial observation that *Ẇ*_B_ during exercise was higher in females than in males (Wanke et al., 1991), this finding has been replicated across several studies (Dominelli, Molgat‐Seon et al., 2015, 2015; Guenette et al., 2007; Mann et al., 2020; Molgat‐Seon et al., 2018; Peters et al., 2021). However, no previous study has assessed the confounding effect of FVC or TLC on sex differences in *Ẇ*_B_. Our results further support the notion that females have a higher *Ẇ*_B_ for a given ${\dot{V}}_{E}$ during exercise and extend this observation by accounting for the effect of FVC. The fact that the effect of sex on the *Ẇ*_B_–${\dot{V}}_{E}$ relationship is not influenced by FVC lends additional support to the contention that male–female differences in airway size and the associated impact on the resistive component of *Ẇ*_B_ is the mechanism that underlies sex differences in *Ẇ*_B_ during exercise.

### 4.6 Conclusion

We examined how FVC impacts the relationship between *Ẇ*_B_ and ${\dot{V}}_{E}$ in healthy males and females. We noted that even after accounting for the effect of FVC, both statistically and by matching males and females based on FVC and age, sex differences in *Ẇ*_B_ persisted. Our findings provide additional evidence supporting the hypothesis that male–female differences in large conducting airway luminal area are the primary physiological mechanism that causes sex differences in *Ẇ*_B_ during exercise.

## AUTHOR CONTRIBUTIONS

All authors were involved in the design of the study. Gracie O. Grift, Jack R. Dunsford, Jasvir K. Dhaliwal and Yannick Molgat‐Seon enrolled participants, conducted data collection and analysed the data. All authors had complete access to all the study data, contributed to drafting and critically revising the manuscript. All authors have read and approved the final version of this manuscript and agree to be accountable for all aspects of the work in ensuring that questions related to the accuracy or integrity of any part of the work are appropriately investigated and resolved. All persons designated as authors qualify for authorship, and all those who qualify for authorship are listed.

## CONFLICT OF INTEREST

None declared.

## Data Availability

The data that support the findings of this study are available from the corresponding author upon reasonable request.
